# Variability and Number of Circulating *csd* Alleles in a Honey Bee Breeding Population After Four Years of Single-Drone Insemination

**DOI:** 10.3390/genes17010086

**Published:** 2026-01-14

**Authors:** Maria Grazia De Iorio, Barbara Lazzari, Maria Cristina Silvia Cozzi, Michele Polli, Giulietta Minozzi

**Affiliations:** 1 Department of Veterinary Medicine and Animal Sciences—DIVAS, University of Milan, 26900 Lodi, Italy; cristina.cozzi@unimi.it (M.C.S.C.); michele.polli@unimi.it (M.P.); giulietta.minozzi@unimi.it (G.M.); 2 Institute of Agricultural Biology and Biotechnology, Consiglio Nazionale delle Ricerche, 20133 Milano, Italy; barbara.lazzari@gmail.com

**Keywords:** *Apis mellifera*, *complementary sex determiner*, *Varroa destructor*, breeding, genetic diversity

## Abstract

**Background**: *Varroa destructor* is the major threat to honey bee health, and selective breeding for resistance traits such as Varroa-sensitive hygiene represents a promising long-term strategy for controlling mite populations. However, breeding programs that rely on highly controlled mating schemes, including single-drone instrumental insemination, may reduce allelic diversity at the complementary sex determiner (*csd*) locus, potentially increasing the production of non-viable diploid males and compromising colony fitness. **Methods**: To evaluate whether *csd* diversity can be maintained under these conditions, we characterized the hypervariable region of *csd* in a selectively bred *Apis mellifera* population subjected to four years of selection. Using a validated de novo assembly pipeline, we reconstructed 43 amino-acid sequences from 33 diploid worker pupae sampled across 13 colonies. **Results**: Seven distinct alleles were identified, five of which were shared among multiple colonies and corresponded to variants already described in the literature, while two were private to individual colonies and novel in the literature. Colony-level frequency data revealed a moderate diversity: the most common allele was detected in nine colonies, with an allelic frequency of 31%. Moreover, the expected heterozygosity of the population was estimated at 0.79. **Conclusions**: Overall, these findings show that *csd* diversity can be partially maintained even under strong selective pressure when multiple maternal lines are retained, and they underscore the importance of incorporating genetic information into breeding decisions to support the long-term sustainability of selective breeding programs.

## 1. Introduction

Honey bees (*Apis mellifera*) are key pollinators that significantly contribute to global food production and ecosystem stability [1,2]. However, managed populations are increasingly exposed to multiple stressors, including pesticide exposure, parasites, pathogens, climate change and habitat loss, which can negatively impact colony survival and genetic diversity [3,4,5,6]. Among these stressors, the ectoparasitic mite *Varroa destructor* represents the most critical biological threat and is widely recognized as the leading cause of colony mortality in temperate regions [7,8]. *Varroa* reproduces within capped brood cells and vectors several viruses, impairing immune function, decreasing worker lifespan, and ultimately leading to colony collapse when unmanaged [9,10,11,12].

Traditional *Varroa* control strategies largely rely on chemical treatments [13], but increasing acaricide resistance and concerns about residues in hive products have increased interest in breeding bees selected for heritable resistance traits [14,15,16,17]. Among the naturally occurring defensive mechanisms, Varroa Sensitive Hygiene (VSH) has proven particularly effective and therefore represents a major target for selective breeding programs [18,19,20,21]. VSH workers detect and remove brood parasitized by reproductive mites during early pupal development, interrupting the mite’s reproductive cycle and reducing the number of viable offspring [22,23]. A phenotypic manifestation of this behavior is the presence of non-reproductive mites inside brood cells, quantified as Suppressed Mite Reproduction (SMR), a widely applied proxy for VSH performance [24,25]. Selection based on SMR has been shown to significantly reduce mite populations and maintain lower infestation levels even when SMR queens are mated with unselected drones [25,26,27,28].

While selective breeding for VSH can substantially enhance colony resilience, such programs often rely on limited founder populations and controlled mating schemes. These practices may inadvertently reduce genetic diversity at essential loci, including the complementary sex determiner (*csd*) gene, which governs sex determination in honey bees [29,30]. Heterozygosity at the *csd* locus leads to female development, whereas homozygosity produces diploid males, which are removed by workers and consequently reduce colony fitness [30,31]. Because loss of allelic diversity at the *csd* locus increases the risk of diploid male production, maintaining high *csd* diversity is critical for the long-term sustainability of breeding populations [32,33].

In this study, we investigate a selectively bred population of *A. mellifera* targeted for high VSH performance, evaluated through the SMR phenotype using a standardized selection scheme involving instrumental insemination, artificial infestation and Harbo test phenotyping. Our aim is to assess whether intensive selection for *Varroa* resistance can be achieved while conserving *csd* allelic diversity, thereby supporting the development of resilient and genetically robust honey bee lines.

## 2. Materials and Methods

### 2.1. Selective Breeding Scheme and Phenotyping

A structured selective breeding program aimed at enhancing resistance to *Varroa* has been operating since 2021, using the Suppressed Mite Reproduction (SMR) phenotype as a quantitative proxy for Varroa Sensitive Hygiene (VSH). In each annual cycle, forty experimental colonies were established in Miniplus hives headed by queens produced through instrumental single-drone insemination (SDI) to generate genetically homogeneous test units composed of supersister workers. Each year, the five highest-performing queens were selected for reproduction. The best-performing queen was dedicated to drone production for SDI, while the remaining four were used to rear daughter queens. Approximately ten larvae were grafted from each of these four queen mothers, producing forty virgin queens for insemination.

To standardize the expression of resistance traits, colonies were artificially infested with *V. destructor*. Mites were collected from external, non-selected colonies and approximately 150 live mites were introduced into each Miniplus colony by placing them on a paper substrate dusted with powdered sugar, which was positioned inside the hive. The sugar attracted worker bees, facilitating contact and transfer of mites onto the bees’ bodies.

Two weeks after infestation, colonies were evaluated for the SMR traits using Harbo test. Capped brood from purple-eye to pre-emergence stages was sampled from one or more frames per colony, and cells were manually uncapped and examined under a stereomicroscope [24]. Mites were classified as reproductive when at least one viable offspring was observed at a developmental stage consistent with the host pupa age. Mites producing only eggs or protonymphs, or whose development was inconsistent with expected timing, were classified as non-reproductive [24,34]. SMR expression was calculated as the proportion of non-reproductive mites relative to the total number of infested cells inspected, with assessment discontinued after 500 cells if fewer than 20 mites were detected.

Based on SMR scores, the highest-performing colonies were selected as queen mothers for the subsequent breeding cycle, and their daughter queens were instrumentally inseminated with semen from drones produced by the single top colony identified in the same evaluation. This integrated pipeline links phenotypic selection with controlled mating to increase *Varroa* resistance while maintaining a standardized genetic structure across experimental units. A comprehensive description of the selection framework is provided in De Iorio et al. [28].

### 2.2. Sampling

Worker samples were collected in 2024 from thirteen colonies belonging to the VSH-selected breeding population (colonies V1–V13), all located within a single apiary in Cremella (LC), Lombardy, Italy, situated in a predominantly rural and agricultural landscape. Four diploid workers were sampled from each colony, with the exception of colony V7, for which only two individuals were obtained, resulting in a total of 50 samples. To ensure accurate colony assignment and eliminate the risk of sampling drifting workers, individuals were collected at the late pupal stage (purple-eye to pre-emergence), while still sealed under brood cappings. Immediately after collection, each pupa was placed individually into a separate 1.5 mL Eppendorf tube containing ethanol and stored at 4 °C until DNA extraction.

All thirteen colonies were headed by queens produced through instrumental SDI using semen from different drones, each originating from the same drone-producing queen. Consequently, workers sampled within each colony were supersisters, sharing identical paternal alleles and approximately 75% genetic relatedness. Due to the SDI design, the theoretical maximum number of *csd* alleles represented within each colony is three: two maternal alleles carried by the queen and one paternal allele contributed by the inseminating drone. Moreover, considering maternal ancestry, the colonies were grouped into two genetic lines: colonies V1–V9 belonging to GL1, and colonies V10–V13 belonging to GL2.

### 2.3. DNA Extraction, Library Preparation and Sequencing

Genomic DNA was extracted from whole pupal tissues preserved in ethanol. Each sample was ground in liquid nitrogen, and DNA extraction was performed using the E.Z.N.A.^®^ Insect DNA Isolation Kit (Omega Bio-tek, Norcross, GA, USA), following the manufacturer’s protocol. DNA concentration was quantified using a Qubit 2.0 Fluorometer (Invitrogen, Carlsbad, CA, USA), and DNA integrity was assessed using the Agilent 2100 Bioanalyzer High Sensitivity DNA assay (Agilent Technologies, Santa Clara, CA, USA).

Sequencing libraries were prepared using the Celero™ DNA-Seq Library Preparation Kit (Tecan Genomics, Redwood City, CA, USA) and sequenced on an Illumina NovaSeq X platform in paired-end 150 bp mode. Base calling and demultiplexing were performed using Illumina BCL Convert v4.3.13.

Read processing and alignment were conducted using the Parabricks v4.4.0 accelerated pipeline. Reads were aligned to the *A. mellifera* reference genome Amel_HAv3.1 (strain DH4; assembly GCF_003254395.2) using GPU-PBBWA-mem v4.4.0.1. Only uniquely mapped reads were retained, and PCR duplicates were marked and removed prior to downstream processing. Sorted and indexed BAM files generated from this workflow were then used directly for HVR sequence extraction and assembly.

### 2.4. Bioinformatic and Statistical Analysis

Sequencing reads corresponding to the *csd* exon 7 region, which contains the hypervariable region (HVR), were extracted using SAMtools v1.15. After trimming and quality filtering, reads were aligned to the *A. mellifera* reference genome Amel_HAv3.1, coordinates: CM009933.2: 11,771,976–11,772,119 [35,36,37]. For each sample, all reads mapping to this interval were isolated and exported as individual FASTA files for downstream reconstruction.

De novo assembly of HVR sequences was performed using CAP3 (parameters: –o 40, –p 90), following the general workflow reported in Paolillo et al. [35] and De Iorio et al. [36]. The resulting nucleotide contigs were then translated into amino acid sequences in all six reading frames using EMBOSS Transeq v6.6.0.0. Only in-frame translations corresponding to full-length protein fragments spanning the conserved region from KI* to EQI, as defined by Bilodeau and Elsik [38], were retained. Assemblies producing more than two protein contigs or containing truncated fragments were discarded. For each individual, the two distinct complete amino acid sequences reconstructed from the contigs were considered to represent the two *csd* HVR alleles expected in diploid workers. To assess allelic novelty and confirm identity, reconstructed HVR amino acid sequences were compared against the NCBInr database using tBLASTn (NCBI, web interface), retaining only matches with 100% query coverage and 100% sequence identity. Additionally, all sequences were cross-referenced with the standardized Amelcsd-HVR nomenclature database to determine correspondence to previously described alleles [38,39]. Reconstructed HVR amino-acid sequences were aligned using ClustalW v2.1 implemented in Jalview (version 2.11.5.1), and the resulting alignment was used to visualize sequence similarity, length variation, and conserved regions among alleles.

To describe allelic distribution, frequencies were calculated in two ways. First, “colony frequencies” were obtained by scoring the presence of each allele across the 13 colonies, reflecting how many colonies carried each variant irrespective of the number of worker samples. Second, because workers from the same single drone-inseminated colony share the same set of *csd* alleles, colonies were treated as independent units for allelic frequency estimation. For each allele, its frequency was defined as the number of colonies in which it was detected divided by the total number of allele occurrences in the dataset. The resulting normalized allele frequencies were then used to compute the expected heterozygosity following Nei et al. [40] as:

(1) $$He=1-\sum_{i=1}^{k}p_{i}^{2}$$

## 3. Results

The hypervariable region (HVR) of the *csd* gene was analyzed in 50 diploid worker bees from 13 colonies. After read processing and alignment to the *A. mellifera* reference genome (Amel_HAv3.1), a total of 1330 reads mapping to the HVR interval were obtained.

From the 50 samples, 43 HVR amino-acid sequences were successfully reconstructed from 33 individuals. Of these, 10 samples yielded both alleles, while in 23 samples only one allele could be successfully reconstructed. In the remaining 17 samples, allele reconstruction was not possible due to insufficient or low-quality read support.

**Figure 1:**
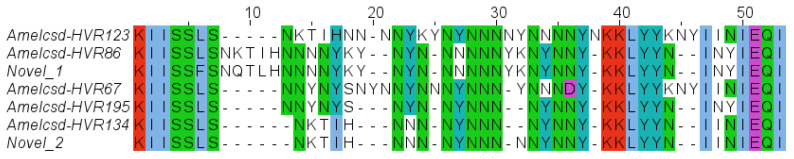
Multiple-sequence alignment of the seven reconstructed csd HVR amino-acid alleles. Colors indicate conserved and variable amino acid residues across alleles.

Across the 43 reconstructed sequences, seven distinct HVR alleles were identified (Table 1). Five alleles were shared among at least two colonies, whereas two alleles were detected only in a single colony. Comparison with the standardized Amelcsd-HVR nomenclature [38,39] revealed that five alleles correspond to previously described variants, while two represent novel alleles, as additionally confirmed through tBLASTn searches against the NCBI nr database (Table 1). A multiple sequence alignment of the reconstructed HVR amino-acid sequences, illustrating sequence length variation and conserved motifs among alleles, is shown in Figure 1.

**Figure 2:**
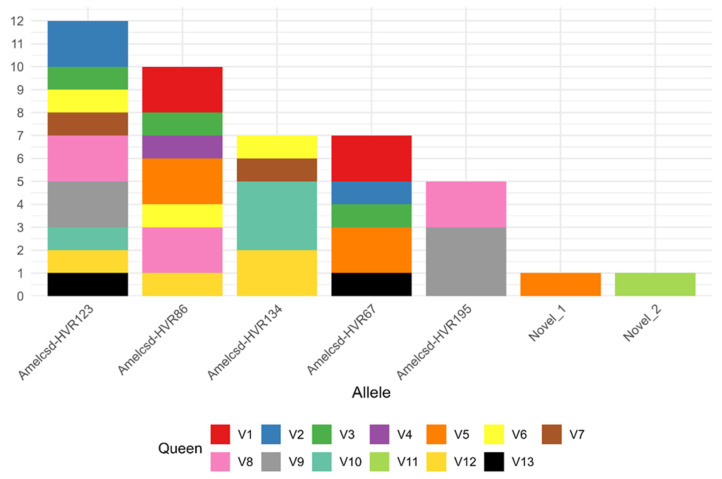
Histogram showing the occurrence of the seven *csd* HVR alleles identified in this study across the 13 colonies. Colors indicate the colony IDs in which each allele was detected. Colonies V1–V9 belong to Genetic Line 1 (GL1), whereas colonies V10–V13 belong to Genetic Line 2 (GL2).

The most frequent allele in the dataset was Amelcsd-HVR123, detected in nine out of the thirteen colonies (69.2%) and present in both genetic lines (Figure 2). Moreover, because workers from the same SDI colony share an identical set of up to three *csd* alleles, allele frequencies were calculated at the colony level rather than at the individual-worker level. For each allele, its frequency was defined as the number of colonies in which it was detected divided by the total number of allele occurrences (*n* = 29). Using this normalization, the allelic frequency of Amelcsd-HVR123 was 31.03% (Table 1), and the expected heterozygosity (H_e_), estimated using Nei’s gene diversity index [40], was 0.79. When H_e_ was calculated separately for the two maternal genetic lines, was 0.78 for GL1 and 0.75 for GL2.

Moreover, when grouped by colony, complete reconstruction of all three expected alleles (two maternal plus the single paternal allele contributed through SDI) was obtained for five colonies. In six colonies, only two alleles were recovered, and in two colonies only one allele was detected (Table 2).

Moreover, when grouping samples based on maternal ancestry, the colonies were grouped into two genetic lines GL1 (colonies V1–V9) and GL2 (coloniesV10–V13). Four alleles (Amelcsd-HVR67, Amelcsd-HVR86, Amelcsd-HVR123, Amelcsd-HVR134) were detected in both genetic lines, two alleles (Novel_1 and Amelcsd-HVR195) were observed only in GL1, and one allele (Novel_2) was found only in GL2.

Finally, consistent with expectations for functional sex determination, all workers analyzed were heterozygous at the *csd* locus, and the two alleles carried by each individual differed substantially in both length and amino acid composition.

## 4. Discussion

Understanding how selective breeding and different controlled mating strategies affect genetic diversity at the *csd* locus is essential for evaluating the long-term sustainability of managed honey bee populations [32,33]. In breeding programs that rely on controlled mating, and particularly on single-drone insemination (SDI), the risk of reducing allelic diversity is substantial, as SDI inherently limits the paternal contribution to a single allele per colony. Because homozygosity at *csd* leads to diploid male production and colony fitness losses, concerns remain that intensive selection may accelerate allele erosion [30,31]. In this study, we assessed *csd* diversity in a selectively bred VSH population maintained under a standardized SDI based selection regime, thereby providing a focused evaluation of diversity dynamics under strong directional selection for *Varroa* resistance.

Using an assembly pipeline previously validated on large datasets [35,36], we reconstructed *csd* HVR alleles from diploid worker pupae collected from 13 colonies. HVR reconstruction was achieved for 33 out of 50 individuals, yielding a total of 43 amino-acid sequences. This output is comparable to those reported in previous work using similarly heterogeneous resequencing datasets [35,36].

In addition to the prior validation of the pipeline used [35,36], the biological coherence of the reconstructed *csd* alleles in the present study is supported by multiple independent observations. First, tBLASTn searches against the NCBInr database, together with cross-referencing against the standardized Amelcsd-HVR nomenclature [38,39], confirmed that five of the seven alleles identified correspond to previously described variants. Second, the number of alleles detected per colony was fully consistent with the genetic constraints imposed by the SDI mating design: each colony is expected to harbor a maximum of three *csd* alleles, corresponding to the two maternal alleles carried by the queen and the single paternal allele contributed by the inseminating drone. Consistent with this expectation, no colony ever displayed more than three alleles.

Moreover, as expected for functionally viable diploid workers, all individuals analyzed were heterozygous at the HVR of the *csd* locus, consistent with the requirement for heterozygosity at this gene to ensure female development [31].

A key finding of this study is the substantial diversity retained at the *csd* locus despite intensive selective breeding. Across the 13 colonies, we identified seven distinct HVR alleles, five of which were shared among multiple colonies and two of which were unique to individual colonies (Figure 2). The presence of private alleles (expected in small populations) also indicates that drift or founder events have not yet eliminated rare variants. However, the most common allele (Amelcsd-HVR123) occurred in 69.2% of colonies, indicating a high but not fixed frequency. The distribution of alleles across maternal genetic lines (GL1 and GL2) further supports the absence of lineage-specific loss: four alleles were present in both lines, suggesting that selection focused on VSH phenotypes did not impose strong unintended bottlenecks at this locus. This pattern is consistent with findings by De Iorio et al. [36], who similarly reported that HVR alleles remained broadly distributed across genetic lines, indicating that substantial *csd* variability can persist within each lineage even after multiple years of selection.

Consistent with previous studies, the seven alleles reconstructed in this population differed markedly in both amino-acid composition and sequence length, ranging here from 38 to 47 amino acids [35,36,38,39,41,42,43,44]. This variability mirrors earlier reports of broad structural heterogeneity, including the extensive variation observed in Italian and European populations, where lengths may span from 27 to over 50 amino acids [35,36,38,41].

Nonetheless, the expected heterozygosity in our population (H_e_ = 0.79) is lower than values reported in other breeding populations, where estimates ranged from 0.90 to 0.96 [36,45], and markedly below the levels observed in large open populations (H_e_ = 0.98) [35]. This outcome is fully consistent with the restrictive mating structure imposed by SDI and the limited number of contributing parental lines. When expected heterozygosity was estimated separately for the two maternal genetic lines, values were further reduced (GL1: H_e_ = 0.78; GL2: H_e_ = 0.75), with the lower value observed in the line composed of fewer colonies. This pattern illustrates how partitioning a breeding population into smaller, related subgroups leads to a reduction in expected heterozygosity, even though observed heterozygosity at the *csd* locus is necessarily equal to 1, reflecting the obligatory heterozygous state required for female development. Importantly, despite these constraints, the level of *csd* diversity retained in this population indicates that selection for *Varroa* resistance has not resulted in a severe loss of allelic richness at this locus. In fact, when our results are compared with those from a closed New Zealand breeding population, in which sixteen alleles were identified across forty-two queens using diploid drones, the diversity observed here appears relatively high in relation to the smaller population size and sampling effort, with seven alleles detected across only thirteen colonies [45]. As expected, the diversity observed here is lower than that reported in geographically broad datasets such as Paolillo et al. [35] or Lechner et al. [46], which reported 88 and 87 alleles, respectively, across large samples covering multiple subspecies and regions.

Notably, in five colonies all three expected *csd* alleles were retrieved, indicating that worker-brood genotyping can reliably capture the complete allele set present within a colony. This finding highlights a practical application of the approach, as it allows breeders to select colonies for controlled matings based not only on phenotypic performance but also on their *csd* allele composition, without sacrificing queens of high breeding value.

## 5. Conclusions

In conclusion, even after four years of single-drone insemination, based on selection for *Varroa* resistance, *csd* diversity in this breeding population has been partially maintained. Although allelic diversity is necessarily lower than that observed in large or unmanaged populations, it remains sufficient for functional sex determination and compares favorably with values reported for other closed or semi-closed breeding systems. These results indicate that directional selection for resistance traits can be compatible with the preservation of genetic diversity at the *csd* locus, particularly when controlled mating strategies are combined with informed breeding decisions that account for both phenotypic performance and lineage structure. Moreover, the ability to reconstruct complete *csd* allele sets from worker brood highlights a practical tool for managing mating compatibility in selective breeding programs.

## Figures and Tables

**Table 1:** Reconstructed amino-acid sequences of the hypervariable region (HVR) of the *csd* gene, including the allele identifier used in this study (ID Allele), the sequence length (L), the colony IDs in which each allele was detected, their frequency among the 13 colonies analyzed, and the allelic frequency. ID Allele corresponds to the standardized Amelcsd-HVR nomenclature when previously described in the literature [38,39], while alleles not matching any published sequence were assigned provisional identifiers (Novel_1 and Novel_2).

HVR Sequences	ID Allele	L	Colony Frequency	Allelic Frequency
KIISSLSNNYNYSNYNNYNNNYNNNYNNNDYKKLYYKNYIINIEQI	Amelcsd-HVR67	46	38.46%	17.24%
KIISSLSNKTIHNNNNYKYNYNNNNNNYKNYNNYKKLYYNINYIEQI	Amelcsd-HVR86	47	53.85%	24.14%
KIISSLSNKTIHNNNNYKYNYNNNNYNNNNYNKKLYYKNYIINIEQI	Amelcsd-HVR123	47	69.23%	31.03%
KIISSFSNQTLHNNNNYKYNYNNNNNNYKNYNNYKKLYYNINYIEQI	Novel_1	47	7.69%	3.45%
KIISSLSNKTIHNNNNYNNNNYNNYKKLYYNIINIEQI	Amelcsd-HVR134	38	30.77%	13.79%
KIISSLSNNYNYSNYNNYNNNNYNNYKKLYYNINYIEQI	Amelcsd-HVR195	39	15.38%	6.90%
KIISSLSNKTIHNNNNYNNNNNYNNYKKLYYNIINIEQI	Novel_2	39	7.69%	3.45%

**Table 2:** Amino-acid sequences of the hypervariable region (HVR) of the *csd* gene reconstructed for the 33 diploid worker bees analyzed. Columns report the genetic line (GL), the colony identifier (ID queen), the individual sample identifier (ID sample), the reconstructed HVR sequences, and the corresponding allele identifier (ID Allele).

GL	ID Queen	ID Sample	HVR Sequences	ID Allele
GL1	V1	2	KIISSLSNNYNYSNYNNYNNNYNNNYNNNDYKKLYYKNYIINIEQI	Amelcsd-HVR67
			KIISSLSNKTIHNNNNYKYNYNNNNNNYKNYNNYKKLYYNINYIEQI	Amelcsd-HVR86
		3	KIISSLSNKTIHNNNNYKYNYNNNNNNYKNYNNYKKLYYNINYIEQI	Amelcsd-HVR86
		4	KIISSLSNNYNYSNYNNYNNNYNNNYNNNDYKKLYYKNYIINIEQI	Amelcsd-HVR67
	V2	5	KIISSLSNKTIHNNNNYKYNYNNNNYNNNNYNKKLYYKNYIINIEQI	Amelcsd-HVR123
		6	KIISSLSNNYNYSNYNNYNNNYNNNYNNNDYKKLYYKNYIINIEQI	Amelcsd-HVR67
		8	KIISSLSNKTIHNNNNYKYNYNNNNYNNNNYNKKLYYKNYIINIEQI	Amelcsd-HVR123
	V3	10	KIISSLSNKTIHNNNNYKYNYNNNNNNYKNYNNYKKLYYNINYIEQI	Amelcsd-HVR86
			KIISSLSNNYNYSNYNNYNNNYNNNYNNNDYKKLYYKNYIINIEQI	Amelcsd-HVR67
		11	KIISSLSNKTIHNNNNYKYNYNNNNYNNNNYNKKLYYKNYIINIEQI	Amelcsd-HVR123
	V4	14	KIISSLSNKTIHNNNNYKYNYNNNNNNYKNYNNYKKLYYNINYIEQI	Amelcsd-HVR86
	V5	17	KIISSLSNKTIHNNNNYKYNYNNNNNNYKNYNNYKKLYYNINYIEQI	Amelcsd-HVR86
		18	KIISSLSNNYNYSNYNNYNNNYNNNYNNNDYKKLYYKNYIINIEQI	Amelcsd-HVR67
		19	KIISSFSNQTLHNNNNYKYNYNNNNNNYKNYNNYKKLYYNINYIEQI	Novel_1
			KIISSLSNNYNYSNYNNYNNNYNNNYNNNDYKKLYYKNYIINIEQI	Amelcsd-HVR67
		20	KIISSLSNKTIHNNNNYKYNYNNNNNNYKNYNNYKKLYYNINYIEQI	Amelcsd-HVR86
	V6	23	KIISSLSNKTIHNNNNYNNNNYNNYKKLYYNIINIEQI	Amelcsd-HVR134
		24	KIISSLSNKTIHNNNNYKYNYNNNNYNNNNYNKKLYYKNYIINIEQI	Amelcsd-HVR123
			KIISSLSNKTIHNNNNYKYNYNNNNNNYKNYNNYKKLYYNINYIEQI	Amelcsd-HVR86
	V7	26	KIISSLSNKTIHNNNNYKYNYNNNNYNNNNYNKKLYYKNYIINIEQI	Amelcsd-HVR123
			KIISSLSNKTIHNNNNYNNNNYNNYKKLYYNIINIEQI	Amelcsd-HVR134
	V8	27	KIISSLSNKTIHNNNNYKYNYNNNNYNNNNYNKKLYYKNYIINIEQI	Amelcsd-HVR123
		28	KIISSLSNKTIHNNNNYKYNYNNNNNNYKNYNNYKKLYYNINYIEQI	Amelcsd-HVR86
			KIISSLSNKTIHNNNNYKYNYNNNNYNNNNYNKKLYYKNYIINIEQI	Amelcsd-HVR123
		29	KIISSLSNNYNYSNYNNYNNNNYNNYKKLYYNINYIEQI	Amelcsd-HVR195
		30	KIISSLSNKTIHNNNNYKYNYNNNNNNYKNYNNYKKLYYNINYIEQI	Amelcsd-HVR86
			KIISSLSNNYNYSNYNNYNNNNYNNYKKLYYNINYIEQI	Amelcsd-HVR195
	V9	31	KIISSLSNNYNYSNYNNYNNNNYNNYKKLYYNINYIEQI	Amelcsd-HVR195
		32	KIISSLSNNYNYSNYNNYNNNNYNNYKKLYYNINYIEQI	Amelcsd-HVR195
		33	KIISSLSNKTIHNNNNYKYNYNNNNYNNNNYNKKLYYKNYIINIEQI	Amelcsd-HVR123
			KIISSLSNNYNYSNYNNYNNNNYNNYKKLYYNINYIEQI	Amelcsd-HVR195
		34	KIISSLSNKTIHNNNNYKYNYNNNNYNNNNYNKKLYYKNYIINIEQI	Amelcsd-HVR123
GL2	V10	35	KIISSLSNKTIHNNNNYNNNNYNNYKKLYYNIINIEQI	Amelcsd-HVR134
			KIISSLSNKTIHNNNNYKYNYNNNNYNNNNYNKKLYYKNYIINIEQI	Amelcsd-HVR123
		36	KIISSLSNKTIHNNNNYNNNNYNNYKKLYYNIINIEQI	Amelcsd-HVR134
		38	KIISSLSNKTIHNNNNYNNNNYNNYKKLYYNIINIEQI	Amelcsd-HVR134
	V11	41	KIISSLSNKTIHNNNNYNNNNNYNNYKKLYYNIINIEQI	Novel_2
	V12	43	KIISSLSNKTIHNNNNYKYNYNNNNNNYKNYNNYKKLYYNINYIEQI	Amelcsd-HVR86
			KIISSLSNKTIHNNNNYNNNNYNNYKKLYYNIINIEQI	Amelcsd-HVR134
		44	KIISSLSNKTIHNNNNYKYNYNNNNYNNNNYNKKLYYKNYIINIEQI	Amelcsd-HVR123
		45	KIISSLSNKTIHNNNNYNNNNYNNYKKLYYNIINIEQI	Amelcsd-HVR134
	V13	49	KIISSLSNKTIHNNNNYKYNYNNNNYNNNNYNKKLYYKNYIINIEQI	Amelcsd-HVR123
		50	KIISSLSNNYNYSNYNNYNNNYNNNYNNNDYKKLYYKNYIINIEQI	Amelcsd-HVR67

## Data Availability

These phenotypes are based on a breeding population used for selection by commercial breeders and have commercial value. Therefore, restrictions apply to the availability of these data, which are not publicly available. The authors can be contacted for a specific request.
